# 100 Ma sweat bee nests: Early and rapid co-diversification of crown bees and flowering plants

**DOI:** 10.1371/journal.pone.0227789

**Published:** 2020-01-29

**Authors:** Jorge Fernando Genise, Eduardo S. Bellosi, Laura C. Sarzetti, J. Marcelo Krause, Pablo A. Dinghi, M. Victoria Sánchez, A. Martín Umazano, Pablo Puerta, Liliana F. Cantil, Brian R. Jicha

**Affiliations:** 1 División Icnología, CONICET-Museo Argentino de Ciencias Naturales Bernardino Rivadavia, Buenos Aires, Argentina; 2 Museo Paleontológico Egidio Feruglio, Trelew, Chubut, Argentina; 3 Grupo de Investigación en Filogenias Moleculares y Filogeografía, Facultad de Ciencias Exactas y Naturales, Universidad de Buenos Aires, Buenos Aires, Argentina; 4 INCITAP and Facultad de Ciencias Exactas y Naturales, CONICET-Universidad Nacional de La Pampa, Santa Rosa, La Pampa, Argentina; 5 Department of Geoscience, University of Wisconsin-Madison, Madison, Wisconsin, United States of America; Geological and Mining Institute of Spain, SPAIN

## Abstract

100 Ma sweat bee nests reported herein are the oldest evidence of crown bees. A new phylogeny for short-tongued bees, calibrated with these nests dated with ^40^Ar/^39^Ar, attests for the first time for a late Albian rapid diversification of bees along with angiosperms. Such hypothesis lacked paleontological support until this study. The new ichnospecies *Cellicalichnus krausei*, which was found along with wasp trace fossils and new beetle trace fossils in the Castillo Formation of Patagonia, represents typical Halictini nests composed of sessile cells that are attached to main tunnels. According to geological, paleosol, paleobotanical, and ichnological data, bees, and angiosperms cohabited in an inland and dry environment comparable to an open dry woodland or savanna, under warm-temperate and semiarid-subhumid climate, in the Southern Hemisphere by the Albian.

## Introduction

The bees play one of the most important roles in our present ecosystems because they are the main pollinators of the dominant flowering plants [1–5]. Also, bees could have played an important role in past ecosystems favoring the diversification of eudicots [5], and forming part of the 125–80 Ma Cretaceous Terrestrial Revolution [6]. The knowledge of its evolutionary history and coevolution with eudicots is critical to understand how our ecosystems operate today and how they can be preserved.

Molecular phylogenetics estimates that the crown group of bees originated approximately 123 Ma (113–132 Ma) concurrently with the rise and expansion of eudicots [5]. However, this hypothesis has not been supported by the fossil record until now. The earliest record of bees, about 98–99 Ma [7], is *Melittosphex burmensis*, which in fact has been disputed as a true bee and belongs to no extant family [5, 8–10]. The oldest crown bee is *Cretotrigona prisca*, which is probably a Meliponinae of about 65 Ma [1, 11], although this age remains controversial. The oldest fossil bee nests attributed to sweat bees (Halictinae) until now, and particularly to Halictini, are *Cellicalichnus dakotensis* from the late Cenomanian of USA [12], and *Cellicalichnus chubutensis* from the Cenomanian of Argentina [13–14]. Other late Cretaceous-Paleogene bee nests were attributed to other tribes of Halictinae [15]. This ichnological evidence provides older records of crown bees than body fossils. However, it has not been considered in the time-calibrated phylogenies proposed, including the latest one [5, 16]. This is done for the first time herein triggered by the discovery of the oldest bee nests, which would be also the oldest evidence of crown bees. Paleoichnological evidence has been only exceptionally considered in phylogenetic approaches, even when architecturally complex traces represent behavioral characters as reliable as the morphological ones found in body fossils, with a plus: they yield information on the behavior and ecology of their producers [15, 17]. Recently, it has been proposed that the attribution as a bee based only on morphological traits of body fossils should be considered as doubtful and that the only feature that distinguishes bees from wasps is the consumption of floral products by larvae [4, 9]. Bee trace fossils provide that type of information.

**Fig 1:**
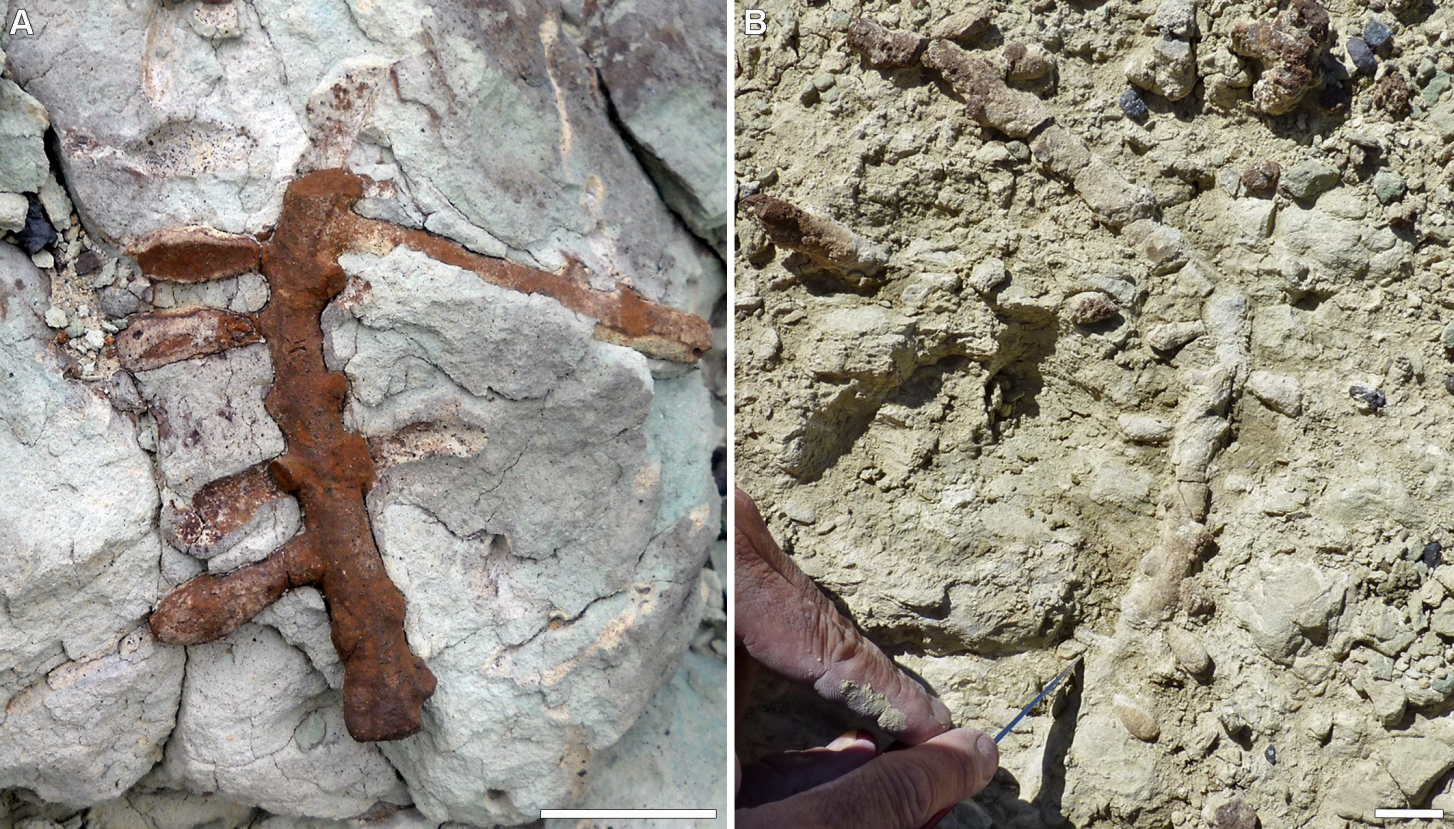
*Cellicalichnus krausei* isp. nov. preserved in a paleosol from the Castillo Formation in Tronador canyon (Argentina). (A) The first specimen found (MPEF-IC 4602, paratype). Scale bar = 2.5 cm. (B) The longest specimen (MPEF-IC 4600, holotype). Scale bar = 2.5 cm.

Here we report a new fossil nest, *Cellicalichnus krausei* isp. nov. (Fig 1), which based on its derived morphology composed of sessile cells attached to main tunnels can be attributed to Halictini as discussed below. They were preserved in a paleosol formed in volcanic ash from the Albian Castillo Formation of Patagonia in Argentina, whose new radiometric age has been calculated for this study in 100.14 ± 0.32 Ma. This new evidence, supported also by other bee fossil nests, allowed us to recalibrate the clade of the short-tongued bees from the recent phylogeny for bees [5]. This provides for the first time hard paleontological evidence to support the hypotheses of the rapid co-diversification of crown bees along with flowering plants during the early Cretaceous. These nests also represent a case of behavioral stasis for sweat bees along 100 m.y. until the present. The paleoenvironment that these oldest bees inhabited was also inferred based on sedimentological, paleopedological, ichnological, and paleobotanical data. This approach yields light and new insights about the paleoecological setting of this early co-diversification of bees and flowering plants in dry environments.

## Materials and methods

### Material and its provenance

The trace fossils examined in this study are permanently deposited in the collection of Ichnology of the Museo Paleontológico Egidio Feruglio (MPEF-IC 4600–4927), Trelew, Chubut province, Argentina. They are publicly deposited and accessible by others in this repository. All necessary permits were obtained for the described study, which complied with all relevant regulations: the Secretaría de Cultura of Chubut Province authorized the collection and loan for the study of the trace fossils (Note n° 114–2017/ D.I.–S.C.). No aspect of the materials or methods of this study needed to be approved by our institutions’ ethical committees.

**Fig 2:**
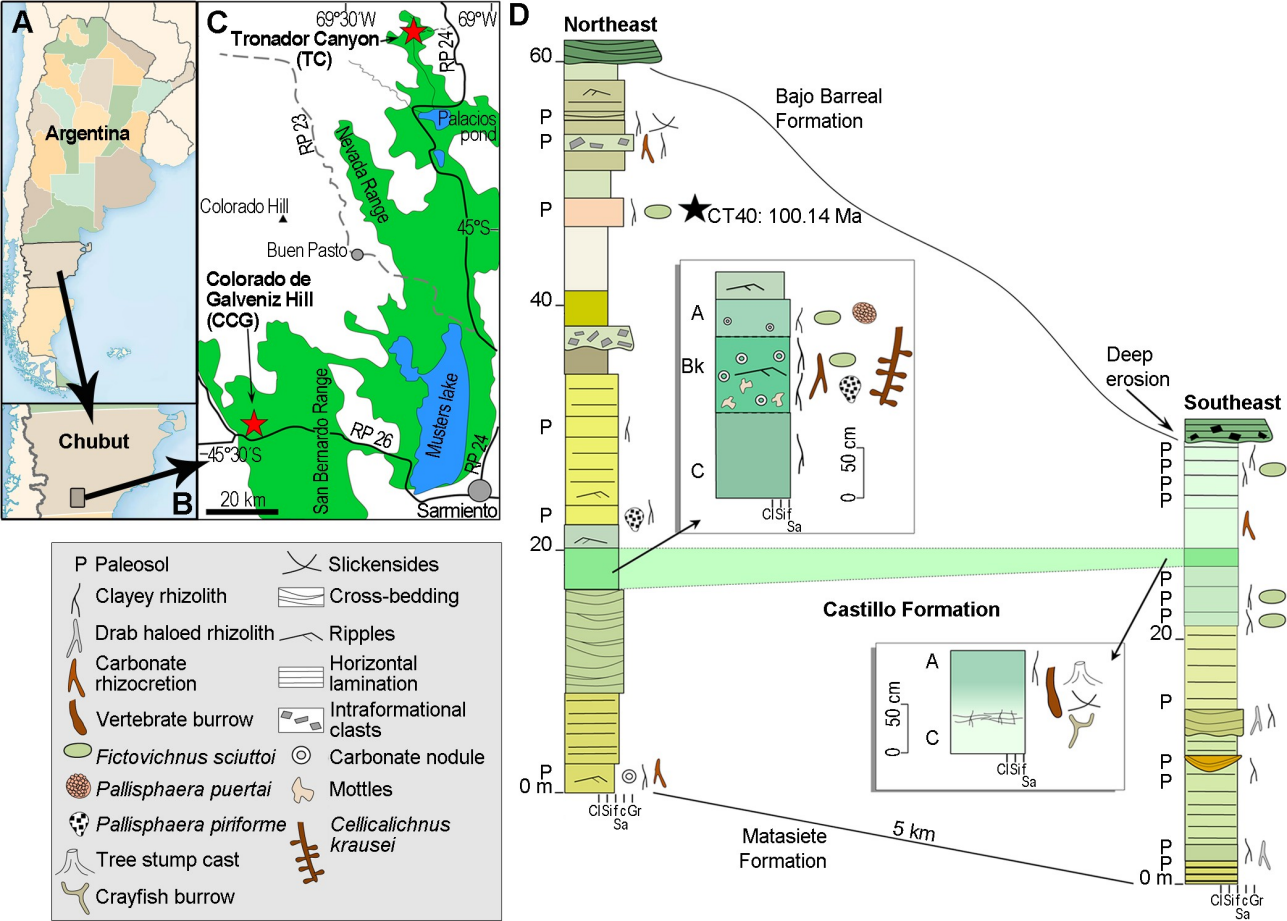
Geographic position and sedimentologic profiles. (A) Location of the studied sites in Argentina. (B) Chubut province. (C) Outcrops of the Cretaceous Chubut Group (green) in the southwest of Chubut. Red stars indicate the two studied sites of the Castillo Formation. (D) Measured sections of the Albian Castillo Formation in the Tronador canyon, north sector of the San Jorge basin. Details of paleosols (P) are shown. The black star indicates the position of the radiometric date presented herein.

Specimens were collected in tuffaceous paleosols of the Castillo Formation (CF), at Tronador canyon (TC) and Colorado de Galveniz hill (CCG), in the southwest of Chubut province, Argentina (Fig 2). The CF is included in a continental succession (Chubut Group), which filled the San Jorge basin during Cretaceous time. The studied areas are located in the west region of this basin, more precisely in the San Bernardo fold and thrust belt. Paleogeographic reconstructions indicate that this region was located in the inland of central Patagonia [18]. The CF is a greenish and pinkish continental succession mostly composed of reworked volcaniclastic sediments. It was formed in fluvial systems during a lapse of increased explosive, Plinian eruptivity that produced large ash-falls from volcanic vents located in the Patagonia Cordillera [19]. At TC and CCG, the CF is 38–60 m and 380 m thick, respectively. The former locality is placed very next to the northwestern basin boundary, where the CF would represent a condensed section [20], while CCG is placed in an intermediate position [21–22]. In the central basin, the CF reaches 2 km in thickness [18].

Based on paleontological data and stratigraphic relationships, the CF was originally considered Aptian [23–24] or Aptian-Albian [18, 25]. Radiometric dates reported previously from the San Bernardo range were Albian-Cenomanian (Ar/Ar 104.8 ± 0.75–97.9 Ma) [26] and middle Albian (U/Pb 104.1 ± 1.4 Ma) [27]. For this study, two new fine-grained tuffs from the upper section of the CF were dated by the ^40^Ar/^39^Ar method. One sample (CT40) comes from the upper part (45 m from the base) of the measured section, above the paleosol containing the nests at the northeastern sector of the TC (Fig 2). The remaining sample (BB18) was obtained 23 m from the base of the CCG section and above the paleosol bearing trace fossils (S1 Fig). Calculated mean ages in plagioclase crystals are nearly coincident in both samples: 100.14 ± 0.32 Ma and 100.13 ± 0.28 Ma, respectively, and are very near to the Albian-Cenomanian boundary. Considering that both samples correspond to the upper section of the CF and that the bee nests occur in the lower section, a late Albian age is inferred for the studied stratigraphic interval.

The CF comprises cross-bedded tuffaceous sandstones and conglomerates with channeled geometry, interbedded with massive or laminated sheet-like deposits of tuffs with accretionary lapilli, tuffaceous sandstones, and mudstones [20, 22]. Fossil woods and leaves, dinosaurs, scarce fishes and mollusks have been recognized in this unit [21, 28]. The CF shows facies assemblages that record fluvial systems composed of channels with different plan-view forms and bars, which were laterally related to floodplains constructed by several mechanisms including sheetfloods, lacustrine sedimentation, debris flows, and preservation of subaerial ash-fall strata [20, 22]. Breaks in sedimentation are registered by paleosols, which are more frequent towards the basin margins, exhibiting low to moderate intensity of bioturbation and a very weak to moderate soil-development degree. Soils formed in fine-grained pyroclastic material, mostly composed of volcanic glass (pumice and shards) and zeolites, along with plagioclase, monocrystalline quartz, volcanic lithic fragments and pyroxene [22]. Accretionary lapilli are commonly recognized in pedogenized massive tuffs.

### Nomenclatural acts

The electronic edition of this article conforms to the requirements of the amended International Code of Zoological Nomenclature, and hence the new names contained herein are available under that Code from the electronic edition of this article. This published work and the nomenclatural acts it contains have been registered in ZooBank, the online registration system for the ICZN. The ZooBank LSIDs (Life Science Identifiers) can be resolved and the associated information viewed through any standard web browser by appending the LSID to the prefix "http://zoobank.org/". The LSID for this publication is *urn*:*lsid*:*zoobank*.*org*:*pub*:*28B74382-B69E-4723-83BB-E25254F6527E*. The electronic edition of this work was published in a journal with an ISSN, and has been archived and is available from the following digital repositories: PubMed Central and LOCKSS.

### ^40^Ar/^39^Ar ages

Sanidine was isolated from samples BB18 and CT40 via crushing, sieving, magnetic and density separations. The sanidine separates were wrapped in aluminum foil and irradiated along in the CLICIT facility at Oregon State University for 65 hours. Single crystal fusion experiments were performed and the Ar isotope analyses were conducted using a Noblesse multi-collector mass spectrometer [29]. All data presented herein are shown with 2σ analytical uncertainties (including J uncertainty) and are calculated relative to a Fish Canyon standard age of 28.201 Ma [30] using the decay constants of Min et al. [31](S2 Fig and S1 Table).

### Attribution of *Cellicalichnus krausei* to bees

The attribution of *C*. *krausei* isp. nov. to an extant group of insects followed the common practice in ichnology, particularly in insect ichnology, for which the more complex traces support more accurate attributions to extant taxa [15, 32–33]. Any hypothesis posing the idea that these nests were constructed by an unknown and extinct group of insects or bees is discarded because it would be much less parsimonious and probable than considering them as constructed by a known group with similar or identical architecture [15]. The morphology of this trace fossil, characterized by rows of sessile cells (i.e. cells are connected directly to the main tunnel, lacking lateral ones), is a derived trait only known among hymenopterans, and particularly among bees. No other insect nests show this morphology [15]. So, the possibility of this nest being of other soil nesting insects is negligible. The sessile cells are a derived and key character that indicates almost with certainty that the nests belong to Halictidae, and particularly to Halictini. In this tribe, the presence of sessile cells is a typical and extended behavioral trait. Sessile cells are exceptionally present in other few species of bees [3, 11, 34]. In *Panurginus albopilosus* (Panurginae) sessile cells are not mentioned but illustrated [35]. The nests of some species of *Lipotriches* (Nomiinae) would also show sessile cells [3]. Besides, sessile cells are present in two Augochlorini (another tribe of Halictinae): *Callochlora* and *Oxystoglosella* [34]. This type of nest is not considered primitive for Augochlorini, but as a derived one [11]. In any case, it would be against the common practice to attribute these nests to the less common and exceptional probable producers, instead of the most common and probable ones (but see more on this in the Discussion below).

### Phylogenetic analysis

In order to understand how this new trace fossil would improve the understanding of bee evolution, a new point of calibration was included on a phylogenetic analysis. Following the methodology used in the last calibrated bee phylogeny [5], a dataset was constructed using sequences from 64 representatives of short-tongued bees: Andrenidae, Colletidae, Stenotritidae, Halictidae, and Melittidae (including Dasypodainae as outgroup [5]), and 4 taxa from Megachilidae and Apidae. Two nuclear ribosomal genes, 18S: small subunit, part; and 28S: large subunit, part, were used to construct the matrix. Sequences were downloaded from Genbank (S2 and S3 Tables). Both genes were aligned with MAFFTv.7 software [36] using the online server on the European Bioinformatics Institute (EMBL-EBI). A final manual alignment was performed using the Bioedit program [37] cutting off regions that could not be aligned with confidence. A total matrix of 68 taxa, each one with 1761 nucleotides, was used (18S: 784 bp.; 28S: 977bp.). The two ribosomal genes were separately analyzed and merged into a single partition. Following the last calibrated bee phylogeny, it was used as an evolutionary model a general-time reversible (GTR), with a proportion of invariable sites (I), and rate variation among sites with four rate categories (G) [5]. A bayesian relaxed-clock log-normal model with five calibration points was used to estimate divergence time. Calibration point 11 (*Electrolictus antiquus*, median 43 Ma, log-normal distribution 95% HPD: 45–60 Ma) [5] was replaced with the record of *C*. *krausei* isp. nov. (100.14 ± 0.32 Ma, normal distribution, node of Halictini-Sphecodini). Two new calibration points were added: *Augochlora leptoloba* (Node: Augochlorini, median: 18.8 Ma, log-normal distribution 95% HPD: 15.97–30.06 Ma) [5] was replaced by the ichnospecies *Corimbatichnus fernandezi*, (54 Ma, normal distribution 95% HDP: 44–60) [15]. Another trace fossil, *Celliforma curvata* [38] was added to calibrate the Diphaglossinae node (Mean: 50.5 Ma, range 48.9–52.1, normal distribution). The other three calibration points used were: *Palaeomacropis eocenicus* (Node: *Macropis* + sister, mean: 53 Ma, normal distribution), *Chilicola electrodominica* and *Chilicola gracilis* (Node: Chilicolini + Xeromelissini, median: 18.8 Ma, log-normal distribution 95% HPD: 15.8–33.32 Ma) and *Heterosarus eickworti* (Node: Melitturgini + Panurgini, median: 18.8 Ma, log-normal distribution 95% HPD: 15.8–33.32 Ma). In all cases, the same mean and statistical distribution used in the last bee calibrated phylogeny were applied [5]. A Yule tree prior was selected, and a root node with a mean of 110 ± 12 Ma, normal distribution [5] was added to avoid as much as possible methodological artifacts associated with relaxed molecular clocks that could pull back node ages [39]. Six independent MCMC bayesian analyses (3 cloned partitions) were run using BEAST 2.x [40] on XSEDE in the CIPRES gateway science research site (https://www.phylo.org/) [41]. Each run was performed with a fixed number of 100 million generations. The parameters for convergence to a stationary state were evaluated with TRACERv. 1.7.1 [42]. In all cases, the first 20 percent of the total topologies were discarded as burn-in. The combined tree files gave a total of 80000 post-burn-in trees for each partition. Using Tree Annotator v.1.8.4 [43] on XSEDE in CIPRES, three maximum clade credibility trees were obtained.

## Results

### Systematic paleontology

Celliformidae Genise, 2000

*Cellicalichnus krausei* Genise and Sarzetti isp. nov. *urn*:*lsid*:*zoobank*.*org*:*act*:*5D4BE8A7-D32E-4B57-89CA-663E7A601E6B*

**Etymology.** Dedicated to the discoverer of the first specimen, our colleague and friend J. Marcelo Krause.

**Fig 3:**
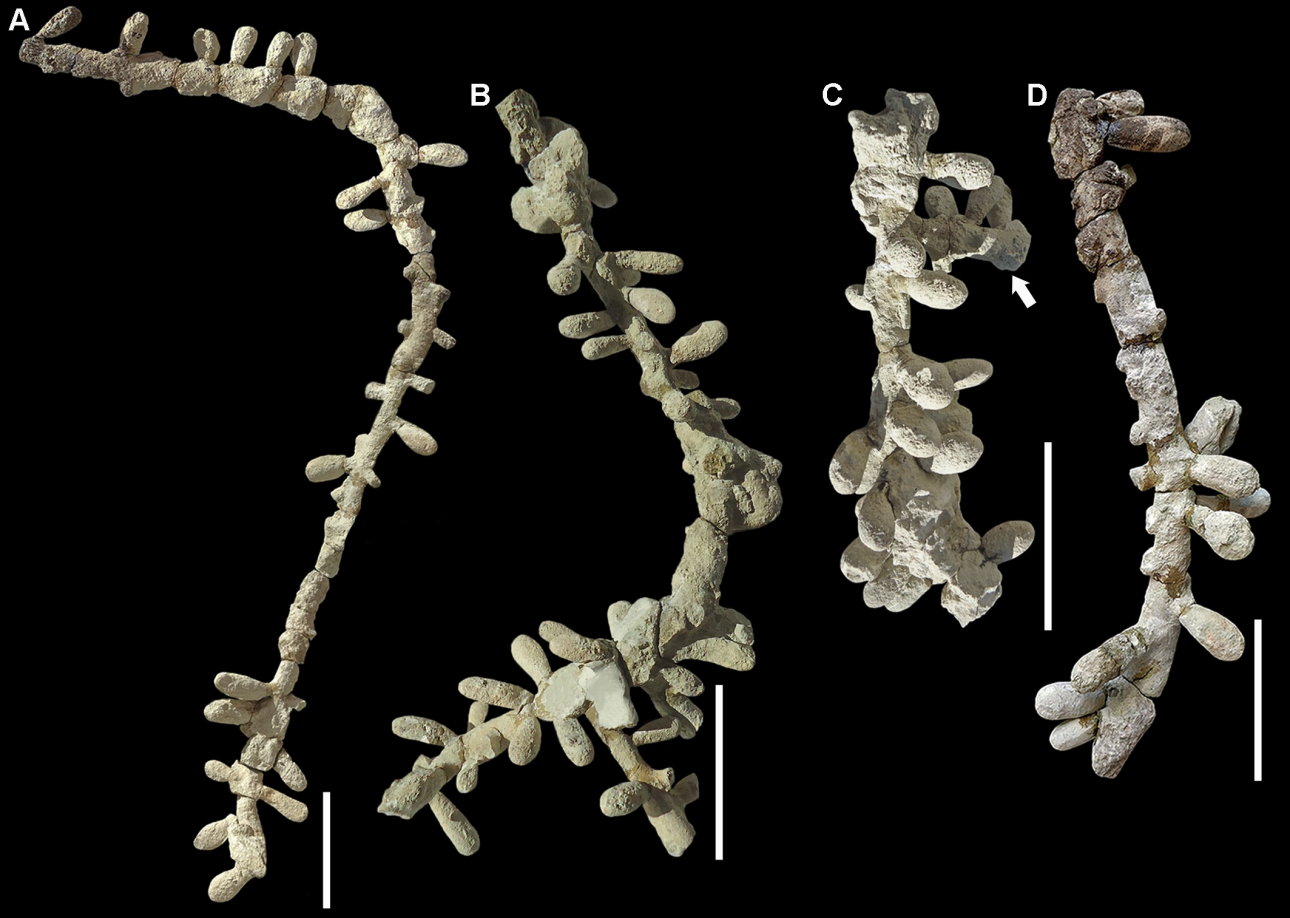
Type material and the most complete specimens of *Cellicalichnus krausei* isp. nov. (A) Holotype (MPEF-IC 4600). (B) Paratype (MPEF-IC 4601). (C) Fragment of a nest showing a lateral tunnel at a right angle (arrow, MPEF-IC 4625). (D) Fragment of a nest showing cells and scars (MPEF-IC 4610). Note the sessile cells in all cases. Scale bars are 5 cm.

**Type material.** Museo Paleontológico Egidio Feruglio, Trelew, Chubut, Argentina; MPEF-IC 4600 (holotype), the longest specimen collected, removed from the matrix and preserved in full relief (Figs 1B and 3A). MPEF-IC 4601 (paratype), the second-longest nest showing a lateral tunnel, removed from the matrix and preserved in full relief (Fig 3B). MPEF-IC 4602 (paratype), the first specimen found, preserved in full relief in the rock matrix (Fig 1A). All from the Castillo Formation, Tronador canyon, Chubut, Argentina.

**Other material.** A total of 15 remains of nests and 9 samples of isolated cells and tunnels (MPEF-IC 4603–4626) from the Castillo Formation, Tronador canyon, Chubut, Argentina.

**Diagnosis.** Specimens of *Cellicalichnus* composed of irregularly distributed, mostly in a plane, tear-shaped, sub-horizontal cells attached directly to inclined shafts, which rarely branch at nearly right angles. The presence of an opposite pair or whorls of cells is occasional. The diameter of shafts is similar to slightly larger than those of cells. Cells show smooth linings and passive fill.

**Comments.** It is an intermediate morphology between *C*. *dakotensis* and *C*. *chubutensis*. The former shows vertical main tunnels and short horizontal ones with opposite pairs of cells and the latter shows the cells arranged in dense whorls. Both the opposite pairs and the whorls of cells are occasionally present in *C*. *krausei*, only in small parts of the tunnels.

**Description.** The longest nest (MPEF-IC 4600) is composed of a tunnel that penetrates the substrate at an angle of 35^o^ showing a bend that divides it in a proximal section, 17 cm long, and a distal one, 35 cm long, the latter at an angle of 140^o^ with the previous one (Figs 1B and 3A). The tunnel is 12 mm wide and 9 mm high. Horizontal cells are distributed mostly in a plane along both sections. Besides the 18 cells preserved in the 52 cm tunnel, there are scars of 13 more cells. They are more grouped at the bottom of the tunnel. No whorl of cells is present and only two pairs of opposite cells are recorded. The other most complete nests show similar features. One of the paratypes (MPEF-IC 4601) is composed of a tunnel that penetrates the substrate at an angle of 60^o^ showing a bend that divides it in a proximal section, 14 cm long, and a distal one, 17 cm long, the latter at an angle of 100^o^ with the previous one. It preserves 38 cells and 7 scars. Most of the cells are distributed in the same plane, particularly in the proximal section, whereas some cells from the deeper section are located in other planes forming a few whorls (Fig 3B). The secondary tunnel, 5 cm long, 10 mm wide, and 9 mm high, branching at a right angle from the distal part of the main tunnel, preserves 3 cells in the same plane and a scar in another one. Most cells are sub-horizontal with their rears slightly inclined downwards. The proximal and distal sections of the main tunnel and the entire secondary tunnel show an inclination of about 60^o^. The other paratype (MPEF-IC 4602) is a short (84 mm) portion of a sloping tunnel preserving 3 cells and 2 scars on one side and 5 scars on the other, where a short (65 mm) lateral tunnel bearing two cell scars arise (Fig 1A). The main tunnel is 11 mm wide and 9–8 mm high, whereas the lateral one is 9–6 mm wide. The specimen MPEF-IC 4625 is a straight tunnel, 14 cm long, preserving 23 cells and scars of another two ones, connected to a short, 8 cm long, lateral tunnel at a right angle, preserving 6 cells (Fig 3C). The main tunnel is 13 mm wide and 10 mm high and penetrates the substrate at an angle of 30^o^. In the main tunnel, the cells are irregularly distributed. Most of them are in the same plane, whereas others are in different planes but without forming whorls. In the lateral tunnel, which branches at a right angle from the main one, the preserved cells are mostly distributed in a single plane. Most cells of the entire nest are sub-horizontal, but a few ones, in other planes, are more inclined. The specimen MPEF-IC 4607 is a tunnel, 96 mm long, preserving the external casts of 5 cells and a secondary tunnel, 55 mm long, with another cell. The main and lateral tunnels are 8–7 mm wide. The specimen MPEF-IC 4624 is a remnant of a nest, which shows a straight and sloping tunnel, 16 cm long, 12 mm wide, and 11 mm high, preserving 17 cells distributed in the same plane, some of them in opposite pairs. The remaining fragments of nests (MPEF-IC 4605–4606, 4608–12, 4615–16, 4618, 4623–24) are tunnels, 215–42 mm long, which preserve 10–1 cells and show the same characters as the more complete nests described above (Fig 3D). Some of them are preserved as full relief casts, whereas others are preserved as external casts in the matrix. Other samples correspond to fragments of tunnels without cells, and isolated cells (MPEF-IC 4603–4, 4613–14, 4617, 4619–22). Taking into account the most complete cells, isolated or connected to tunnels, they are tear-shaped, 24–19 mm long (*n =* 154), 11–9 mm wide (*n* = 161), 10–7 mm high (*n* = 171) and the neck is 8–5 mm in diameter (*n* = 173).

Pallichnidae Genise, 2004

*Fictovichnus sciuttoi* Genise *et al*., 2007

**Examined material.** Forty-eight specimens from Cerro Colorado de Galveniz hill (MPEF-IC 4627–4674) and thirty seven specimens from Tronador canyon (MPEF-IC 4675–4711), all from the Castillo Formation of Chubut, Argentina.

**Fig 4:**
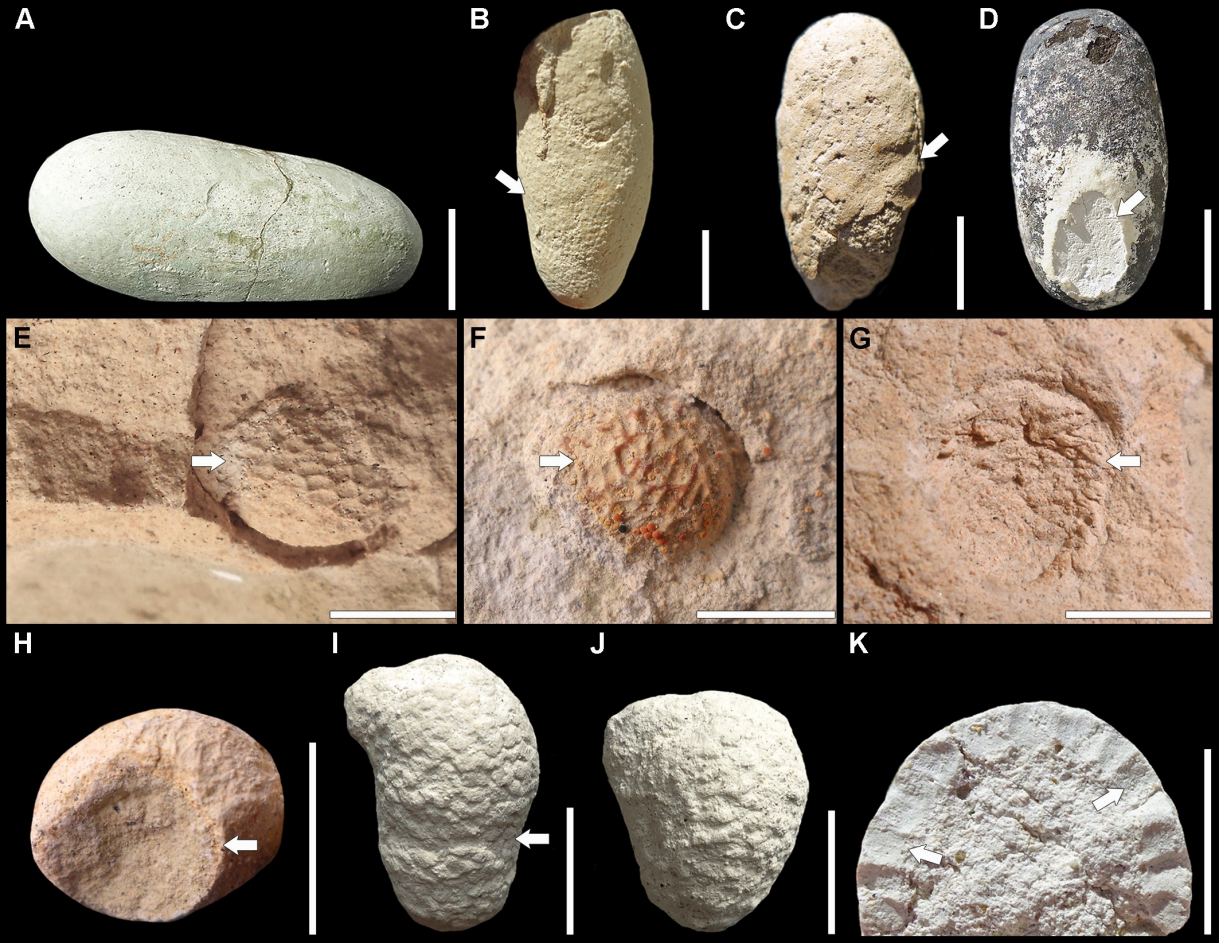
Other insect trace fossils found along with the bee nests in the Castillo Formation. *Fictovichnus sciuttoi* (A–D). (A) Lateral view of a specimen showing the flattened outline (MPEF-IC 4669). (B) Specimen preserving a helical surface morphology composed of fine ridges (arrow, MPEF-IC 4698). (C) Specimen showing rough ridges (arrow, MPEF-IC 4700). (D) Specimen with a flat oval area in one extreme (arrow, MPEF-IC 4683). *Pallisphaera puertai* isp. nov.(E–H). (E) Holotype in the matrix showing the casts of pellets (arrow, MPEF-IC 4712). (F) Paratype in the matrix showing the casts of pellets (arrow, MPEF-IC 4713). (G) Wall of a specimen attached to the matrix (MPEF-IC 4725). Note the pellets (arrow). (H) Specimen with an emergence hole (arrow, MPEF-IC 4718). *Pallisphaera piriforme* isp. nov. (I–K). (I) Holotype showing pellets and the lateral protuberance (MPEF-IC 4732). Note the piriform shape and the tight constriction between the two spheroids (arrow). (J) Ovoid specimen (MPEF-IC 4734, paratype). (K) Longitudinal section of a specimen showing cylindrical pellets (arrows, MPEF-IC 4824). Scale bars are 10 mm.

**Description.** Ellipsoid to ovoid chambers mostly preserved as internal casts horizontally to sub-horizontally oriented in the paleosol (Fig 4). The diagnostic helical surface composed of fine to rough ridges is present in 26% of the specimens (Fig 4B and 4C), whereas the diagnostic flat oval area is present in 21% of the specimens (Fig 4D). Specimens are 42–15 mm long (*n* = 42), 20–6 mm wide (*n* = 76), and 17–6 mm high (*n* = 67). A total of 79% of the specimens shows a flattened outline (Fig 4A).

Coprinisphaeridae Genise, 2004

*Pallisphaera* Genise igen. nov. *urn*:*lsid*:*zoobank*.*org*:*act*:*88DE2197-EFA2-447E-B31B-FFE2A50A65C7*

**Etymology.** Derived from the Greek *palla* meaning ball, and *sphaira* meaning sphere. The combination refers to a sphere whose wall is composed of little balls (pellets).

**Type ichnospecies.** *Pallisphaera puertai* isp. nov.

**Diagnosis.** Spherical to sub-spherical or pear-shaped to ovoid chambers, which are surrounded by a discrete wall composed of smooth, rounded to rhomboid pellets.

**Comments.** It differs from *Coprinisphaera* because of the wall composed of pellets and the lack of an egg chamber and from *Castrichnus* because of the lack of concentric rings in the internal surface of the pellets and the rounded to meniscate pellets in the filling of the chambers.

*Pallisphaera puertai* Genise isp. nov. *urn*:*lsid*:*zoobank*.*org*:*act*:*65DC8879-8022-470B-81E8-3DD1AF030ABA*

**Etymology.** Dedicated to the discoverer of the first specimen and friend, Pablo Puerta.

**Type material.** MPEF-IC 4712 (holotype), a specimen in the matrix showing the casts of pellets in the filling (Fig 4E). MPEF-IC 4713 (paratype), another specimen similar to the holotype (Fig 4F). Both from the Castillo Formation, Cerro Colorado de Galveniz, Chubut, Argentina.

**Other material.** Fourteen specimens from Cerro Colorado de Galveniz hill (MPEF-IC 4714–4727) and four from Tronador canyon (MPEF-IC 4728–4731), all from the Castillo Formation of Chubut, Argentina.

**Diagnosis.** Spherical to sub-spherical *Pallisphaera* showing the wall composed of small rhomboid pellets in some cases arranged in rows parallel to the equator.

**Description.** Specimens are mostly preserved as spheroidal casts showing the concave casts of pellets on their surface, and separated by a thin space from the matrix that represents the wall (Fig 4E and 4F). When the wall is preserved, it is composed of rhomboid pellets (Fig 4G). The equatorial diameter is 17–10 mm (*n =* 17), but in some cases the outline is somewhat elliptical, being one of the axes about 1 mm larger than the other. The height is 16–10 mm (*n =* 10). All specimens in which it was possible to measure wide and height (*n =* 8) show a flattened outline, representing the height 70–90% of the width. The wall itself or the space representing it is 1–2 mm thick (*n =* 10). Rhomboid pellets, 2 x 1 mm, are oriented with the long axis parallel to the equator. One specimen shows on top a rounded hole, 8–9 mm in diameter, which would be compatible with an emergence hole (Fig 4H). Infillings are structureless.

*Pallisphaera piriforme* Genise isp. nov. *urn*:*lsid*:*zoobank*.*org*:*act*:*CA30C2D1-92A4-46BB-914E-5F71D5A07FD0*

**Etymology.** After its pear-shaped shape.

**Type material.** MPEF-IC 4732 (holotype), a complete specimen showing pellets and protuberance (Fig 4I). MPEF-IC 4733–34 (paratypes), two specimens similar to the holotype (Fig 4J). All from the Castillo Formation, Tronador canyon, Chubut, Argentina.

**Other material.** One hundred and ninety-three specimens from the Castillo Formation, Tronador canyon, Chubut, Argentina (MPEF-IC 4735–4927).

**Diagnosis.** Pear-shaped to ovoid *Pallisphaera* showing the wall composed of adjacent rounded to elliptical, small pellets. Specimens are vertically oriented with the thinner end facing down. Some specimens may show a lateral protuberance near the top.

**Description.** Specimens *in situ* look like inverted drops composed of two attached spheroids of different sizes. The smaller one faces down. In the more piriform specimens, the constriction between the two spheroids is tighter (Fig 4I), whereas in the ovoid ones the constriction is gentle (Fig 4J). The height is 25–13 mm (*n =* 140), but half of the specimens is 21–18 mm high. The equatorial diameter of the larger sphere (upper part) is 17–9 mm (*n =* 174), but half of the specimens is 14–13 wide. The equatorial diameter of the smaller sphere (lower part) is 12–6 mm (*n =* 150), but 60% of the specimens are 10–9 mm wide. The wall is 3–1.5 mm thick (*n =* 68) and the pellets are 2–1 mm large (*n* = 50). In a few specimens, the lateral outline shows an undulated pattern (Fig 4I). Pellets are cylindrical in longitudinal section, which is observable in broken and weathered walls (Fig 4K). In a few specimens, the pellets seem to be arranged in diagonal rows. About 35% of the specimens show a sub-cylindrical to rounded lateral protuberance near the top, 8–3 mm wide and 5–2 mm high (*n* = 16). In four specimens the wall shows a rounded discontinuity on top suggesting an emergence hole. Infillings are structureless.

### The soil where the sweat bees nested

The sweat bee nests were found in the lower section of the Albian Castillo Formation (CF) at Tronador canyon (TC). They occur inside a tuffaceous, bright green paleosol that is exposed, with some lateral variations, along several kilometers in the TC. In the northeastern part, where the bee nests were found, this paleosol occurs 17 m above the base of the CF, in fine tuffaceous sandstone with moderately-altered glass shards, not eroded at the top. It shows three horizons. The surface one (A horizon) is 0.4 m thick, yellowish-green in color, and includes small, common light-grey carbonate nodules (2–3 mm) and thin (1–2 mm) clayey rhizoliths. The subsurface (Bk) horizon, 0.8 m thick, preserves isolated current ripples and is slightly lighter and more calcareous and indurated than the A horizon due to micrite cement. It also shows thicker (4 mm) and more frequent clayey rhizoliths, similar carbonate nodules, larger dark-red carbonate rhizocretions extending sub-vertically for 15 cm, and pale greyish-pink mottles probably related to root structures (root drab haloes). In thin-sections, the groundmass is dark (partly isotropic), slightly calcareous, with iron nodules and lacking highly birefringent streaks (undu-calciasepic microfabric). The lower (C) horizon is greyish-green, massive, 0.9 m thick, with non-calcareous matrix and sparse clayey rhizoliths. All rhizoliths are thin, branched and lack taproots. The upper two horizons include *F*. *sciuttoi*, *P*. *puertai*, and *P*. *piriforme* (Fig 4). Bee nests reach up to 1.1 m depth from the top of the paleosol, but their segments with cells occur in the lower 60 cm (Bk horizon). At the south, southwestern and northwestern parts of the TC, the bright green paleosol lacks bee nests, is thinner and less developed. It shows vertebrate burrows cemented by carbonate, crayfish burrows, slickensides and fine rhizoliths mainly arranged horizontally at some decimeters from the top. Large, badly preserved fossil tree stump casts, showing partial carbonate cementation, were also recognized in this paleosol in the southeastern area. Weak-moderate calcareousness suggests that original soil pH was near-neutral or slightly alkaline. Preserved relict bedding (ripples), absence of both textural differentiation and diagnostic horizons, and persistence of glass shards in the paleosol bearing the bee nests indicate relative short to intermediate time for soil formation. Likewise, the absence of voids suggests weak modification of the parent material. The volcaniclastic mineral is responsible for the isotropic microfabrics, commonly found in andic soils. The abundant volcanic shards and crystals allowing classify this paleosol as an Andisol, probably an ustic Vitrand considering the degree of calcification.

### Phylogenetic analysis

**Fig 5:**
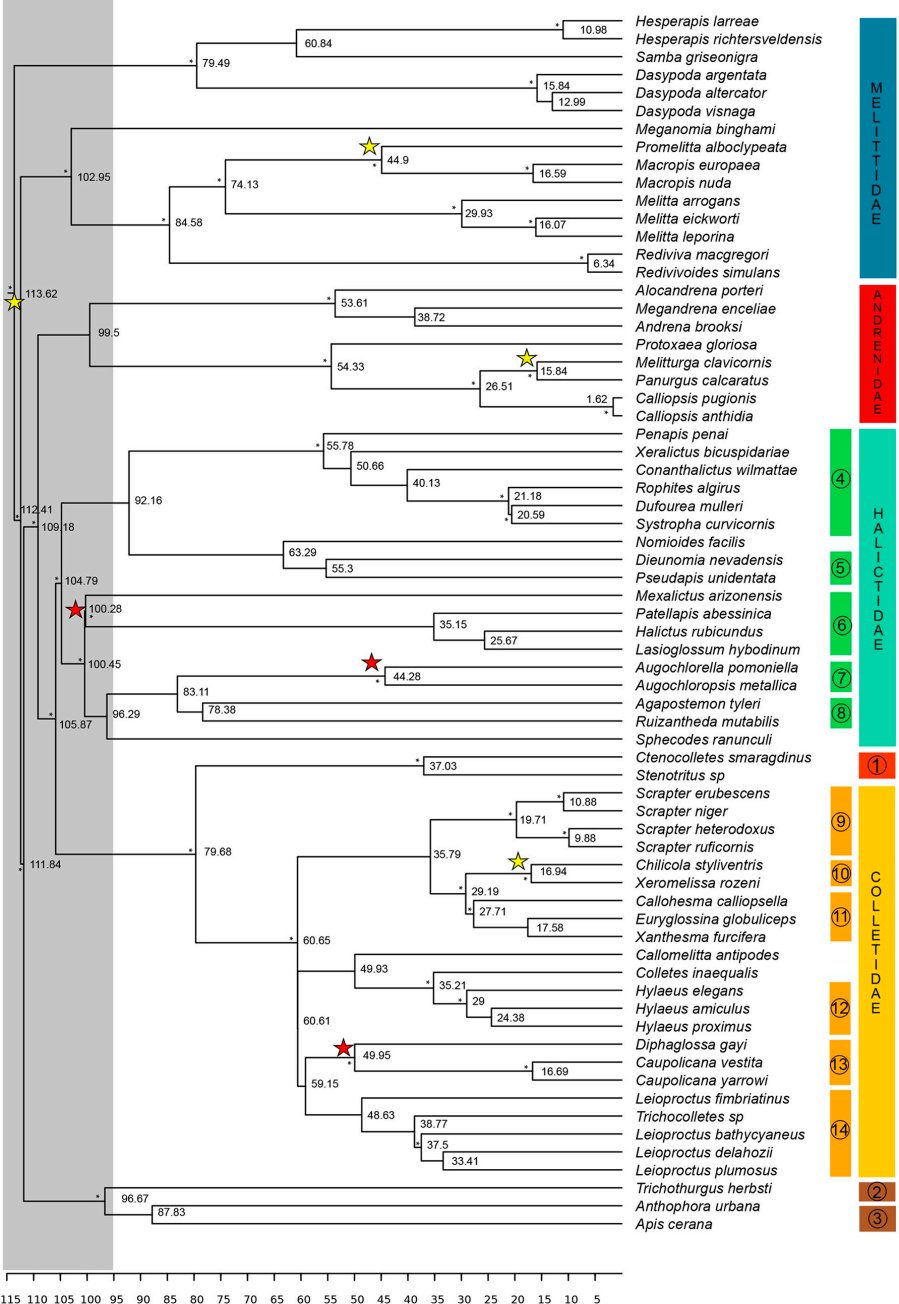
Maximum clade credibility tree obtained from all data. Numbers on the phylogram indicate node ages in Ma. Asterisks on nodes indicate posterior bayesian probabilities >0.9. Calibration points are shown as stars on nodes of groups that they cluster: red stars are trace fossils and yellow stars are body fossils. The latter were already used in previous analyses (except the calibration for root node). Grey area from root to about 95 Ma indicates the Early Cretaceous fast cladogenesis of most bees (this study) and Eudicots [44]. Circled numbers indicate the following taxa: 1-Stenotritidae; 2-Megachilidae; 3-Apidae; 4-Rophitinae; 5-Nomiinae; 6-Halictini; 7-Augochlorini; 8-Caenohalictini (6+7+8+Sphecodini = Halictinae); 9-Scrapterinae; 10-Xeromelissinae; 11-Euryglossinae; 12-Hylaeinae; 13-Diphaglossinae; 14-Neopasiphaeinae.

The three maximum clade credibility trees obtained in this research, which included new five calibration points (see Methods) showed almost identical topologies, with nonsignificant differences among them. The phylogram of the merged partition of both ribosomal genes is shown in Fig 5.

All principal monophyletic groups from the last proposed phylogeny [5] were recovered (families and subfamilies), as well as hierarchical relationships among them. The root node age, which is considered the origin of extant bees, is estimated at 113.6 Ma. Since then, and during about 35 Ma, the tree shows a multiple and fast cladogenesis giving rise to the appearance of the most important groups as follows: Melittidae vs. sister 112.4 Ma; long-tongued bees vs. short-tongued bees 111.8 Ma; Andrenidae vs. Halictidae + Colletidae + Stenotritidae 109.2 Ma; Halictidae vs. Colletidae + Stenotritidae 105.9 Ma, and inside them, Halictidae 104.8 Ma, Andrenidae 99.5 Ma, and Colletidae vs. Stenotritidae 79.9 Ma. In particular, Halictidae undergoes six cladogenesis events in less than 27 Ma corresponding to almost all subfamilies and even tribes (Rophitinae, Halictinae, Halictini, Caenohalictini, Sphecodini). On the side of long-tongued bees, diversification occurs in the same period of time. Megachillidae vs. Apidae diverged about 97 Ma. After these major "evolutionary chain" events, the cladogenesis shows a deceleration period in many groups and gradual changes. Colletidae presents a stasis period followed by gradual cladogenesis among groups that started 60 Ma. The same pattern is observed for Stenotritidae, although it could be an artifact because it is underrepresented in the sample.

## Discussion

### Evolutionary history

The radiation of angiosperms is one of the most discussed topics in plant evolution [45], but no matter the approach, it is usually related to the origin and diversification of bees and bee pollination [1–2, 4–5, 45–46]. Probably, pollination evolved from pollen consumption [47] and early Cretaceous angiosperms were pollinated by generalized insects other than bees, which already visited gymnosperms [4, 48–49]. However, at present, the derived angiosperms grouped in the Eudicot clade, representing about 75% of the diversity of flowering plants, are mostly bee-pollinated [5]. Eudicots are supposed to have arisen in the Barremian (130–125 Ma) based on the first appearance of tricolpate pollen grains [4–5, 44, 50–53]. Pollen adaptations to pollinating bees, such as pollen clumps due to sticky grains, were reported from middle Cenomanian rocks [54]. Fossil flowers showing features compatible with bee pollination are younger, from the Turonian (ca. 90 Ma) [45, 54–55]. Ancestral angiosperms would have been favored by an increasingly specialized pollination [56]. The origin of bees based on wasp body fossils [4] and molecular phylogeny [5] has been calculated in 125–120 Ma. However, the oldest crown bee body fossil known until now is only about 65 Ma [1, 11]. Nevertheless, this topic has been discussed [5].

So, in the absence of Barremian–Albian crown bee body fossils and flowers showing adaptations for bee pollination, which is the paleontological support for the hypothesis that proposes a common origin for bees and eudicots 125 Ma?, and which for the early Cretaceous rise of flowering plants pollinated by bees? The fossil bee nests *C*. *krausei* provide part of this evidence placing crown bees in the late Albian of Patagonia. The presence of bees in southern South America may be related to the rapid diversification and abundance increase of angiosperms since the middle Albian [57–58]. Recent phylogenies of bees showing a slow and constant rate of cladogenesis along Cretaceous–Cenozoic times [5] were in contrast with the rapid diversification proposed for the major lineages (orders) of angiosperms during the early and mid-Cretaceous [44]. The new phylogeny for short-tongued bees presented herein, which includes the oldest bee nests of *C*. *krausei* isp. nov. and other two bee trace fossils as calibration points (Fig 5), offers answers for the above questions and support for the rapid coevolution of bees and flowering plants during the Early Cretaceous. According to the ichnological evidence, the minimum age for the Halictini node was moved back here almost 40 Ma. The node for Augochlorini was moved from 30 Ma to 44.3 Ma, and the addition of *Celliforma curvata* as calibration point shows that the minimum age for Diphaglossinae is at least 10 Ma earlier than that considered until now [5]. As a result, the topology obtained shows that there was a rapid bee diversification around 100 Ma as expected according to proposals that could not be supported by evidence until now.

Regarding the attribution of these nests with sessile cells to Halictini and its consequence in the obtained cladogram, it is critical to take into account their derived condition. Based on the common pattern with sphecoid wasps, it was established that ancestral nests of bees were composed of a main burrow showing lateral burrows, each one ending in a single cell, and that sessile cells are later modifications of this ancestral pattern [3]. The same is true for Halictinae in particular, the ancestral type is considered to be a nest where cells are located at the end of lateral tunnels [34, 59]. So, although the nests are 100 Ma, the possibility of them being of an ancestral type is unlikely, they represent a derived morphology. Accordingly, if in any case we attribute *C*. *krausei* to the less probable producers, such as particular genera of Panurginae, Nomiinae or derived Augochlorini, instead of the more probable Halictini, the consequence of using derived nests for re-calibrating the phylogeny would have a similar result in the cladogram’s topology: the rapid diversification of groups around the age of these nests.

This new evolutionary scenario can be explained as a series of co-evolutionary events between flowering plants and bees. The fast cladogenesis episodes can be envisaged as an ecological race to conquest new niches, both for plants and bees. In sum, this phylogenetic analysis is congruent in terms of ages with a guild or diffuse co-evolution, where a trait may evolve in several species in reciprocity with the evolution of a trait in other species.

Trace fossils provide not only calibration points in phylogenetic analyses. They are valuable information that must be taken into account in order to find a better hypothesis to explain the history patterns of the distribution and diversity of extant species. They also provide insights on the paleoenvironments that these bees and the angiosperms that provide pollen for their nests inhabited.

### Paleoenvironment of the oldest crown bees

The soil where the Albian sweat bees built their nests developed in the distal sector of a floodplain. In TC, ancient streams of the Castillo fluvial system were small, frequently unchannelized and carrying volcaniclastic materials. Pedogenesis of sediments was recurrent but during short lapses, indicating unstable landscapes, with frequent and brief interruptions in the deposition. Immature paleosols preserved at TC indicate that sedimentary condensation was minimal, including the areas next to basin margins. The original soil formed in fine-grained volcanic ash redeposited by sheetfloods, and then buried by new sheetfloods, probably at an early stage of the successional colonization. It was thick (more than 2.5 meters) and slightly calcareous. The deep and clayey fills of rhizoliths, along with ferrous oxides, indicate unimpeded free drainage. The deep reach of root traces and scarcity of waterlogging features also indicate profound water-table. Iron depleted drab mottles in the subsurface horizon were probably related to the anaerobic decay of roots during early burial [60]. Burial gleization would have also changed the original color of the soil into bright green. The mentioned pedogenic features of the paleosol, including the asepic microfabric (see Methods), point out to a weak soil-development degree and stable drainage. In consequence, these bees were early colonizers taking advantage of extensive and fresh deposits of ash in an early stage of pedogenesis. It is noticeable that bee nests only occur in the drier northeastern sector, but not in the south were crayfish burrows and horizontal rhizoliths of the same paleosol indicate more waterlogged conditions due to topography. The paleosols of the same CF at the locality of Colorado de Galveniz hill (CCG, Fig 2), whose radiometric age obtained for this study (100.13 ± 0.28 Ma) is virtually the same as that obtained for TC (100.14 ± 0.32 Ma), show the same ichnofauna of the southern section of CT, where bee nests are absent (S1 Appendix; S1 Fig and S2 Fig). This evidence suggests that bees preferred better-drained sectors of the landscape to nest, congruently with the known ecological preferences of bees for dry areas [3, 15, 61–62].

Which were the possible sources of pollen and the vegetation cover of these soils? The CF provided fossil leaves from Cachetamán and Melillán I hills and Nahuel canyon in the San Bernardo range, including eudicots [28, 63–65]. The Cachetamán fossil flora shows low diversity and includes several angiosperms, with some ferns and Cycadales [28]. These assemblages are dominated by a single leaf morphotype of an undetermined taxon [65], whereas recognizable angiosperm taxa mainly comprise Lauraceae and eudicots [28, 64]. Undetermined wood fragments were frequently found in the CF at several localities [22, 65]. Late Albian-Cenomanian tricolpate pollen from diverse and primitive eudicots was reported from the lower section of the unit overlying the CF [66]. The presence of eudicots, represented by leaves in the same formation, is a fulfilled condition of the Castillo paleoenvironment to support the presence of bees, which are pollen dependent. In the paleosol where the bee nests occur, the root traces are common, and they are fine, deep penetrating, and lacking taproots, indicating prevalent low plants, presumably herbs and shrubs [67]. The possible stump casts suggest the presence of trees in the southern part of the canyon, where no bee nests were found. Accordingly, the paleolandscape inhabited by the bees was dominated by herbs and shrubs, probably leaving patches of bare soil where the bees nested, with the presence of trees in neighboring areas, which perhaps were more vegetated and moist. The possible scenarios are that bees nested in a dry soil gathering pollen from eudicots growing right there, as many extant bees do, or that the pollen was obtained from the eudicots growing in more vegetated close areas.

Which could be the paleoclimatological conditions? Calcareous accumulations in the Castillo paleosols (*i*.*e*. nodules, rhizocretions, and dispersed carbonate) are suggestive of dry conditions during pedogenesis. Calcite cementation by deep burial diagenesis is discarded because observed replacive and displacive textures and carbonate concentrations in distinct horizons. The carbonate is congruent with the abundance of authigenic analcime in the CF [68], who inferred a semiarid climate during deposition time. This ustic soil moisture-regime corresponds to semiarid-subhumid regions with a mean annual rainfall between 300–700 mm [69]. Clay-filled rhizoliths and calcareous rhizocretions suggest short to moderate seasonality in precipitation [60]. By the Albian, the TC area in central Patagonia was near a paleolatitude of 60^o^ S, within the belt of warm temperate climate [70]. This is partly congruent with the tropical to subtropical distribution of extant Lauraceae, which were found as fossils in the CF. These plants generally live in humid areas, although some species are adapted to semiarid conditions. Castillo fossil floras were interpreted as a vegetal community that took advantage of unsteady environments, which were warm and dry according to leaf morphology [71]. Cycads and bennettitaleans that were frequent by this time show marked adaptations to seasonally dry conditions [72]. Warm conditions also prevailed in southern Patagonia during the late Albian according to pollen grains [73–74]. According to the global reconstructions, biomes from this mid-latitude region included: dry-deciduous and moist-evergreen closed forests, and dry savanna-like environments with sparse trees [75]. The last one is more compatible with the landscape for the CF according to the data presented herein. Bee nests (*C*. *krausei*), wasp cocoons (*F*. *sciuttoi*), and coleopteran pupation chambers (*P*. *puertai* and *P*. *piriforme*) occurring in paleosols with calcareous accumulations are compatible with the *Celliforma* Ichnofacies, which indicates reduced plant coverage resulting in a sunny soil surface under semiarid to arid climates [15]. Regarding this ichnofauna, the ichnospecies of *Pallisphaera* igen. nov. represent the oldest evidence of soil pellet production and utilization by insects.

In summary, geological information, paleosol features, paleobotanical data, and ichnofacies suggest that the Albian Castillo environment at TC developed in an inland region of central Patagonia, comparable to an open dry woodland or savanna-like environment with sparse trees. This habitat had a warm-temperate and semiarid-subhumid climate, with a probable mean annual precipitation of 300–700 mm. Bees would have selected to nest in well-drained floodplain deposits in their early stage of pedogenesis or patches of bare soil. They could have searched for pollen of eudicots growing in this soil or in the neighboring moister and vegetated sectors as shown by the lateral variation of the nesting soil. Such Albian paleoenvironment from Patagonia for crown bees is congruent with former hypotheses proposing that bees would have originated in the xeric interior of Gondwanaland during the Early Cretaceous, and that bees diverged from wasps in the Southern Hemisphere [2, 4, 61–62]. Such scenario contrasts with the original paleoenvironments proposed for angiosperms from the northern hemisphere. During the Barremian–Cenomanian, North American angiosperms would have been ruderal components of coastal, poorly drained environments, such as deltas, estuaries, swamps, and levees [76–77]; whereas European ones first grew in lakes and wetlands and then they became understory components of upland and semiarid floodplain forests [78]. The case presented herein records, for the Southern Hemisphere, the colonization of drier environments by angiosperms during their rapid Albian diversification, perhaps favored by the preference of bees for xeric environments.

## Supporting information

S1 Appendix Geologic setting of the Castillo Formation at Cerro Colorado de Galveniz hill. (DOC)

S1 Fig Measured section of the Albian Castillo Formation (Chubut Group) in Colorado de Galveniz hill. (TIF)

S2 Fig 40Ar/39Ar ideograms of single crystal fusion data from samples BB18 and CT40. (TIF)

S1 Table Complete Ar isotope data and analytical information. (XLS)

S2 Table Accession numbers and complementary data of partial sequences downloaded for 18 ribosomal subunits. (XLSX)

S3 Table Accession numbers and complementary data of partial sequencies downloaded for 28 ribosomal subunits. (XLSX)
