# Supplementary material for: 100 Ma sweat bee nests: Early and rapid co-diversification of crown bees and flowering plants
Source: PLoS One. 2020 Jan 29;15(1):e0227789. doi: 10.1371/journal.pone.0227789 (PMC6989152; doi:10.1371/journal.pone.0227789)
Supplement: S1 Appendix — (DOC) [file pone.0227789.s001.doc]

**100 Ma sweat bee nests: early and rapid co-diversification of crown bees and flowering plants**

Jorge F. Genise, Eduardo S. Bellosi, Laura C. Sarzetti, J. Marcelo Krause, Pablo A. Dinghi, M. Victoria Sánchez,

Aldo M. Umazano, Pablo Puerta, Liliana F. Cantil, Brian R. Jicha

**S1 Appendix.** **Geologic setting of the Castillo Formation at Cerro Colorado de Galveniz hill.**

The Cerro Colorado de Galveniz (CCG) section was originally attributed to the Bajo Barreal Formation [1], but recent observations allow reassigning this interval (122 m thick) to the upper part of the Castillo Formation (S1 Fig). The new isotopic dates presented here, 100.13 ± 0.28 Ma and 100.14 ± 0.32, obtained in CCG and Tronador canyon respectively (S2 Fig and S2 Table), support the correlation of both sections. Paleosols from the CCG section formed on sheet beds of tuffaceous sandstones and fine-grained tuffs, which commonly bear accretionary lapilli. They are very weakly to weakly developed, and exhibit relict bedding, carbonate nodules, and trace fossils [1]. Usually, ash-fall deposits become the stronger developed paleosols. Five of them include *Fictovichnus sciuttoi.* Four of these paleosols are greenish-grey in color and present two horizons with relict lamination. The upper horizon of the more developed examples shows columnar and medium-size granular peds. The lower horizon is mostly unstructured. Scarce to common, fine (2–3 mm) to medium-size (5 mm) rhizoliths, along with crayfish burrows, occur in both horizons. *Fictovichnus* is also present in very-weakly developed paleosols, formed in massive, tuffaceous medium-grained sandstones or a matrix-supported tuffaceous conglomerate with volcanic pebbles. Only a few rhizoliths and probable crayfish burrows are recognized in these cases. *Pallisphaera puertai* occurs in a more developed paleosol along with *Fictovichnus sciuttoi* and crayfish burrows. This light brown to greyish-pink paleosol shows three horizons. The surface one shows coarse blocky peds, abundant clayey rhizoliths, and *Pallisphaera puertai*, *Fictovichnus sciuttoi*, and crayfish burrows. The subsurface horizons are massive and present scarce rhizoliths. According to the geometry and internal facies organization of channel sand bodies, the fluvial system of the CCG area was single-channeled or meandering and probably braided in the upper section [1]. Intermittent ash-falls mantled the topography and were frequently reworked by confined and unconfined flood events. Overbank sheetfloods and fewer debris-flows were the main process of aggradation in floodplains. The weak development of the CCG paleosols suggests an unstable landscape mainly due to common depositional events and subordinated erosional episodes. The paleosols showing redoximorphic features (*e.g*. light-grey mottles and drab-haloed root traces) and crayfish burrows indicate waterlogging conditions at least temporally. *Fictovichnus sciuttoi* in the same horizons had to be constructed when the soil was unsaturated because the trace makers are air-breathers.

**Supplementary References**

1. Umazano A, Bellosi ES, Visconti G, Melchor R. Detecting allocyclic signals in volcaniclastic fluvial

successions: facies, architecture and stacking pattern from the Cretaceous of central Patagonia, Argentina. J S Am Earth Sci. 2012;40:94–115.
