# Supplementary figures and images for: 100 Ma sweat bee nests: Early and rapid co-diversification of crown bees and flowering plants
Source: PLoS One. 2020 Jan 29;15(1):e0227789. doi: 10.1371/journal.pone.0227789 (PMC6989152; doi:10.1371/journal.pone.0227789)

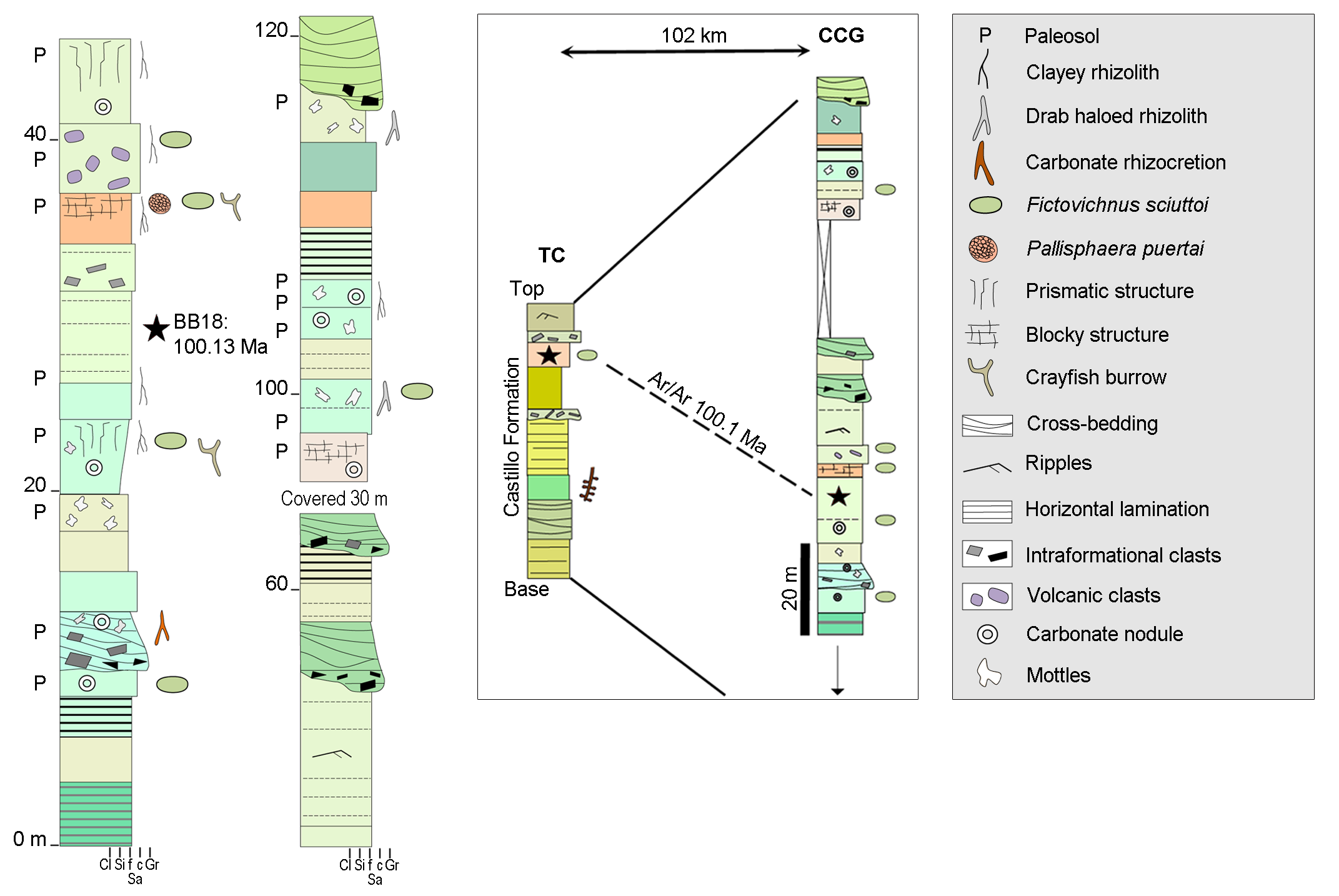

Supplement: S1 Fig — (TIF) [file pone.0227789.s002.tif]

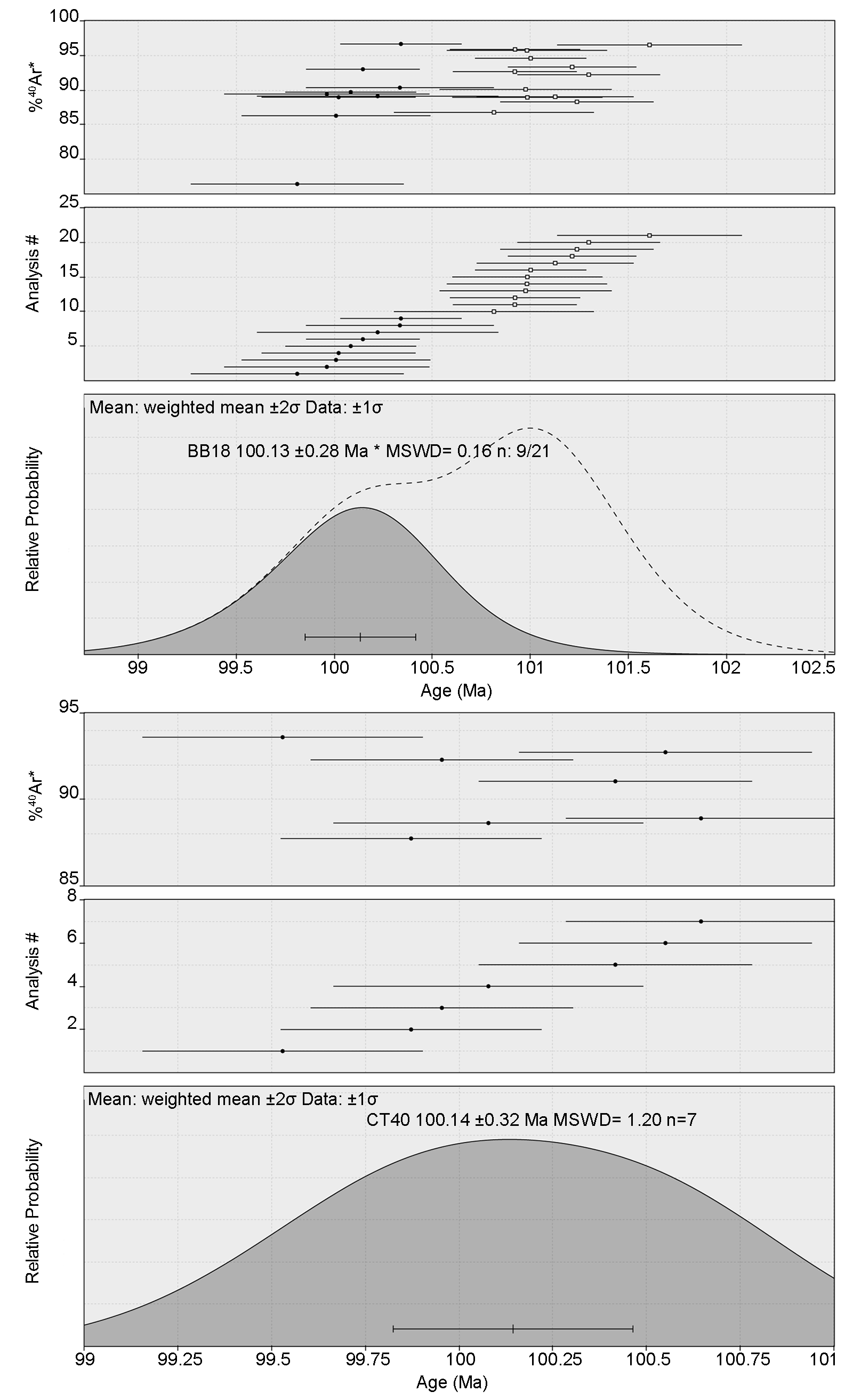

Supplement: S2 Fig — (TIF) [file pone.0227789.s003.tif]
